# How Ethical Leadership Shapes Employees’ Readiness to Change: The Mediating Role of an Organizational Culture of Effectiveness

**DOI:** 10.3389/fpsyg.2019.02493

**Published:** 2019-11-14

**Authors:** Dina Metwally, Pablo Ruiz-Palomino, Mohamed Metwally, Leire Gartzia

**Affiliations:** ^1^Faculty of Commerce and Business Administration, Helwan University, Helwan, Egypt; ^2^Faculty of Business Administration, University of Castilla–La Mancha, Cuenca, Spain; ^3^University of Deusto, Deusto Business School, Bilbao, Spain; ^4^Faculty of Business Administration, University of Deusto, Bilbao, Spain

**Keywords:** ethical leadership, organizational culture of effectiveness, organizational change, readiness to change, organizational culture

## Abstract

Today’s organizations are operating in a highly competitive and changing environment that pushes them to continuously adapt their organizational structures to such environment. However, the success of change initiatives may face a barrier in the response of employees, especially when they lack readiness to change. While leadership can shape the culture of an organization and a culture of effectiveness can help increase employees’ readiness to change, ethical leaders, who serve as a guide and offer support, can also make a difference by reducing uncertainty. Yet existing research on the role of ethical leadership in the enhancement of the employees’ readiness to change is practically non-existent. Far less is the research that analyses the mechanisms that ethical leadership can use to foster employees’ readiness to change. This study aims to investigate whether the ethical leadership of middle–lower echelons influences on employees’ readiness to change positively (H1) and if this relationship is mediated through shaping an organizational culture of effectiveness (H2). Using data from 270 direct reports of middle–lower managers in public foreign trade Egyptian companies, the findings reveal that ethical leadership enhances employees’ readiness to change and that this impact is partially mediated by an organizational culture of effectiveness. Thus, with these findings, new light is shed on the positive role of ethical leadership and the mechanisms it uses to enhance employees’ readiness to change.

## Introduction

Today’s organizations are operating in such a highly dynamic and competitive environment that they need to undergo continuous change. However, most change projects fail to achieve the expected results (Beer and Nohria, 2000; Sirkin et al., 2005), and people’s attitudes are a likely reason for this outcome (Eby et al., 2000; Holbeche, 2006). Some people may welcome change, viewing it as a chance to draw benefits and improve their status in the organization; others, however, view it as a threat and display negative attitudes toward it (Vakola, 2014). In the latter case, people are said to be resistant to change. This resistance might be due to their inability to adjust their behavior, skills, and commitment to meet the new requirements; they may not possess skills related to a readiness to change. Successful change requires readiness to change, as it is a critical factor in bringing about effective implementation of change (Vakola, 2014). As George and Jones, (2001, p. 420) state, “organizations only change and act through their members.”

Individual readiness to change thus plays an important role in every instance of organizational change (Oreg et al., 2011) and appears to be critical in successfully implementing changes in organizations (Jones et al., 2005). This concept is similar to the “unfreezing” concept introduced by Lewin (1951) to refer to the process by which beliefs and attitudes about a pending change are altered in a way that change is perceived as a necessity, and likely to be successful. While theorizing readiness to change in this way has a high level of acceptance (see Choi, 2011), it overemphasizes personal beliefs about the appropriateness of the change in the organization (Armenakis and Bedeian, 1999) and underemphasizes the personal capacities of employees and their willingness to make change efforts. However, the latter elements are a clear indication that the employee is truly ready to change. If an employee is not willing to change, then their adaptation to the change can be limited, thus undermining the success of the implementation of any change in the organization (Soumyaja et al., 2011). In fact, scholars increasingly emphasize involvement and commitment-to-change conceptualizations to refer to this concept (Fedor et al., 2006; Foster, 2010; Vakola, 2013, 2014).

Individual readiness to change is thus defined in terms of reactions toward the change, where the person has confidence in his/her abilities to manage it (Vakola, 2014) by accepting, embracing, and adopting a particular plan to purposefully alter the *status quo* (Rafferty et al., 2012). In Vakola’s words (2013, p. 97), for example, a person is ready to change when he/she “will start or continue being engaged in behaviors associated with change such as support and participation,” which requires that this person “has confidence in his/her own ability to succeed in change.” Thus, taking into consideration these insights from the literature, employees’ readiness to change will be conceptualized in the current study as the extent to which employees have confidence in their own abilities to succeed in change, and are psychologically or physically prepared to participate and be involved in making the change work.

As an individual’s readiness to change is so critical for organizational change to be successful, the identification of its antecedents has become an issue of interest among practitioners and scholars during the last decade (Holt et al., 2007; Choi, 2011; Zayim and Kondakci, 2014). Drawing on Choi (2011) and Holt et al. (2007), these antecedents are typically grouped in terms of context (e.g., organizational culture, leadership), content (e.g., extent, favorableness, and appropriateness of the change), and process (e.g., successful history of change and positive experiences in previous change projects and fairness of the change process). Factors related to the individual are also important antecedents (e.g., change self-efficacy), but several previous research studies (i.e., Stanley et al., 2005; Devos et al., 2007) have suggested that individual factors appear to be far less important than situational variables in predicting an individual’s readiness to change. In fact, for employees to be ready to change, previous studies have revealed the critical influence of the context (Holt et al., 2007), including leadership (Choi, 2011). In particular, transformational leadership has been highlighted as a critical antecedent of readiness-to-change-related outcomes (Caldwell et al., 2009; Michaelis et al., 2010). Because transformational leaders are characterized for articulating a challenging and attractive future vision of the organization as well as for inviting their employees to challenge the *status quo* (Belschak et al., 2015) these leaders are highly likely to enhance the readiness to change of their employees. In effect, these leaders create the vision and institutionalize the change efforts (Tichy and Devanna, 1990) and are more likely than others to be proactive and coach the change, which is critical to prepare employees for change efforts (Armenakis et al., 1993). However, some previous research reveals low levels of variance explained by this type of leadership in some change-related employee outcomes (i.e., change commitment, Yu et al., 2002), which suggests that other elements could also be important in this regard. For example, trust in leaders – intimately related to ethical behavior and ethical leadership (Brown et al., 2005; Stouten et al., 2012; Ng and Feldman, 2015) – is also important to ensure readiness to change (Walker et al., 2007), which suggests that the ethical dimension of the leader could make a difference in helping boost this valuable individual outcome in organizations. Thus, other more ethics-focused forms of leadership approaches may also capture significant variance in predicting such an important employee outcome. Hoch et al. (2016), for example, found that ethics-rooted leadership approaches such as authentic and ethical leadership show similar correlations as transformational leadership with a wide variety of positive employee outcomes (e.g., trust in supervisor, engagement, and job satisfaction). Furthermore, their meta-analytic study found that the more emphasis leaders put on ethics, the stronger their ability to predict positive outcomes (Hoch et al., 2016). Ng and Feldman (2015) also demonstrate that ethical leadership, even in the presence of transformational leadership, is significantly positively related to task performance of employees. Thus, there is the possibility that ethical leadership can play an important role in predicting a valuable outcome in the workplace such as readiness to change, which until now has been practically unexplored.

In effect, a review of the literature reveals that perceiving managers as trustworthy and having faith in their intentions (Vakola, 2014), which is likely to occur when employees are led by ethical leaders (Kalshoven et al., 2011, 2013), can underlie employees having a stronger readiness to change (Choi, 2011). However, research has not explicitly addressed the role of ethical leadership in promoting employees’ readiness to change in organizations. In studies on ethical leadership, only Sharif and Scandura (2014) focused on change, although they did not evaluate the influence of ethical leadership on employees’ readiness to change. In fact, existing research connecting change and leadership has failed to investigate the impact of leadership on change outcomes (Battilana et al., 2010; Myeong-Gu et al., 2012), with studies being more focused on the role of leaders in supporting change (Babalola et al., 2016). However, ethical leaders are, among other things, trustworthy, fair, and people-oriented, and provide ethical guidance (Kalshoven et al., 2011, 2013). They encompass a number of critical features that can reduce the stress and turmoil faced by employees in uncertain and changing times (Sharif and Scandura, 2014). Because stress makes employees develop negative attitudes toward change (Vakola, 2014), ethical leaders may have a positive impact on employees’ readiness to change. Such an impact may also occur indirectly, through shaping the culture of their organizations in a way that favors readiness to change. Leaders determine the aspects in which the culture of their organizations emphasizes most, which ultimately shapes the behavior in the workplace (Schein, 1992), so the idea that ethical leaders could foster readiness to change through shaping the culture of their organizations is underpinning and fills an important void in the literature.

Leaders constitute primary sources of information about salient attributes of the environment (Zohar and Luria, 2005), and play an important role in shaping the culture within the organization (Schein, 1992), a concept that is related but distinct to organizational climate (Ehrhart et al., 2014). The organizational climate describes the shared perceptions of those aspects of the work environment (i.e., policies, practices, and procedures) that inform members about which behaviors will be rewarded, expected, and supported (Reichers and Schneider, 1990; Schneider et al., 2013). The organizational culture instead concerns the shared basic, implicit assumptions (i.e., taken-for granted beliefs about how things should be in the organization that reside below the surface), beliefs, and values that are taught to newcomers as the proper way to think and feel, and that guide the behavior within the organization (Schein, 1992). Thus, while the emphasis in the organizational climate is on tangible policies, practices, and procedures as the causes of people’s experiences, the emphasis in the organizational culture is on the values, beliefs, and assumptions that are implicit in all these mechanisms (Schneider et al., 2013). Leaders, with their espoused values, behavior, and actions, play an important role in shaping both aspects, but may be more important in shaping the system of shared values, beliefs, and assumptions that helps direct employees’ decisions and behaviors within the organization (Schein, 1992). In fact, the measurement of organizational culture has typically focused more on values (Jones et al., 2005) than on artifacts (i.e., the visible and perceptible language, materials, and behaviors in an organization; Schein, 1992).

Thus, by choosing the organizational level of analysis to conceptualize the organizational culture in this study, leadership (either from the upper, middle, or lower echelons) will be considered as helping to embed their beliefs and values into employees’ shared understandings (cf., Schaubroeck et al., 2012). Indeed, leadership is intimately linked to communicating and inspiring values in others (Hood, 2003), and this process is highly likely to be effectuated through embedded mechanisms, both primary (i.e., deliberate role modeling, disciplining, and coaching) and secondary (i.e., organization structures, procedures, and formal statements). Furthermore, these values are more than likely to be fostered because of their usefulness in the past in helping organizations to adapt themselves to external problems and to solve internal integration issues (cf., Schein, 1992). Thus, it is of no surprise that leaders typically become transmitters and drivers of values, beliefs, and assumptions concerning the most important issues faced by employees in gaining organizational effectiveness (Sashkin, 2012).

In this sense, ethical leaders have distinctive characteristics that can have a special influence on shaping an organizational culture of effectiveness, conceptualized as the shared assumptions, beliefs, and values that affect employees’ attitudes and behaviors in a way that drives effectiveness. For example, servant leaders, who practice an ethical form of leadership (Hoch et al., 2016), have an important positive impact on team effectiveness (De Hoogh and Den Hartog, 2008; Hu and Liden, 2011). Team effectiveness, in turn, is usually seen in organizations where the organizational culture fosters organizational change (McNabb and Sepic, 1995). As such, for organizations to be effective in terms of change, shaping an organizational culture of effectiveness that emphasizes aspects, such as dealing with change, working in teams to achieve goals, customer orientation, and the strength of these shared beliefs and values, may be very helpful (Sashkin and Rosenbach, 2013). Such a culture might be more aligned with change objectives which, according to existing research (Eby et al., 2000; Jones et al., 2005), should encourage employees’ readiness to change. This leads us to suggest that ethical leaders could encourage employees’ readiness to change through shaping an organizational culture of effectiveness. Yet, existing research has not addressed any of these issues, so the question of whether ethical leaders foster employees’ readiness to change, and whether an organizational culture of effectiveness mediates this relationship, is an intriguing research gap to fill.

The principal research objective is therefore to explain the role of ethical leadership in encouraging employees’ readiness to change. To this end, this study examines the direct positive effect of ethical leadership, and the mediating effect of organizational culture of effectiveness in this relationship. These efforts advance previous research that has indicated that employees’ readiness to change is positively related to factors such as trust in management (Shah, 2014; Vakola, 2014), support from management (Kirrane et al., 2017), empowerment of employees (Li et al., 2016), and good leader–employee relationships (Shah and Shah, 2010). In addition, by investigating these relationships, this study helps expand the set of positive outcomes of ethical leadership. Also, this investigation is based on an Arab cultural context (i.e., Egypt), where ethical leadership research is lacking. In ethical leadership research, studies using Western societies abound (Brown and Treviño, 2006; Resick et al., 2011), yet the Arab context has been scarcely explored (e.g., Demirtas and Akdogan, 2015; Arar et al., 2016). However, the cultural context can affect how employees react to leadership perceptions (Fu et al., 2007) and could shape the relationship between ethical leadership and its outcomes (Oc, 2018). Thus, by offering findings in a non-Western society like Egypt – that professes high levels of power distance, collectivism, avoidance uncertainty, restraint, and short-term orientation (Hofstede Center, 1967–2010) – this study may offer compelling insights concerning the context-sensitivity or universality of ethical leadership theory (Brown and Treviño, 2006; Kalshoven et al., 2011), particularly on the basis of the relationship that is predicted in this study between ethical leadership and employee readiness to change.

**FIGURE 1:**
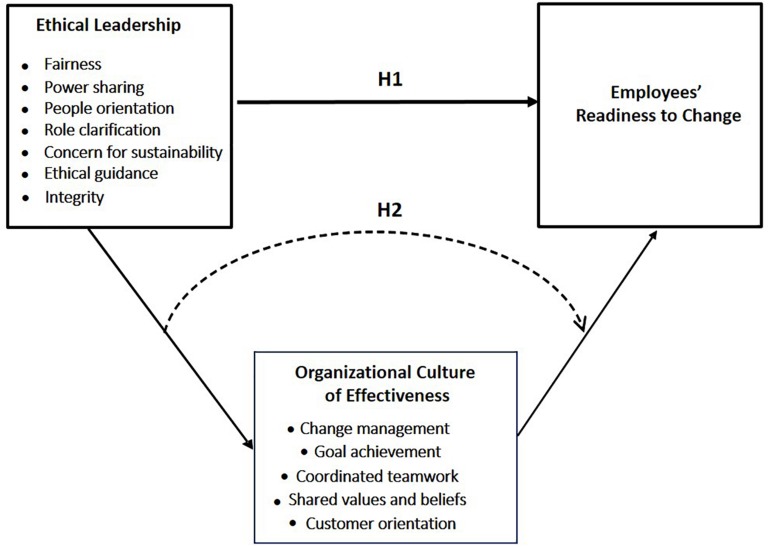
Research model and hypotheses.

This study also advances previous research by investigating the mediating effect of organizational culture of effectiveness on this relationship, and thus by explaining how or why ethical leadership predicts or causes employees’ readiness to change. Although previous research has indicated a positive relationship between ethical leadership-related approaches and team-organizational effectiveness (e.g., ethical leadership, De Hoogh and Den Hartog, 2008; servant leadership, Hu and Liden, 2011), the relationship between ethical leadership and organizational culture of effectiveness has yet to be studied. However, some aspects of an organizational culture of effectiveness (i.e., teamwork; Sashkin and Rosenbach, 2013) enhance employees’ readiness to change (Rodriguez et al., 2016), and could help explain how ethical leadership positively relates to employees’ readiness to change. Thus, the current study will offer new insights into how to succeed in times of organizational change. Using the Egyptian society, this study will also contribute to the literature in Arab societies as well as in countries with similar cultural characteristics. For managers, this study is particularly relevant; it provides new knowledge and strategies to help encourage readiness-to-change-related tendencies in the workplace. Figure 1 summarizes the research model.

### Theoretical Framework

#### Antecedents of Employees’ Readiness to Change: A Brief Literature Review

According to Choi (2011) and Holt et al. (2007), the literature on antecedents of employees’ readiness to change has been developed around four mainstream areas: (1) context, (2) content, (3) process, and (4) the individual. Each of these areas, and its development, has provided important results and advances regarding how to shape readiness to change among employees.

Regarding the area of research related to individual-based factors, a wide range of personal traits have been identified as potential antecedents of readiness-to-change-related outcomes. Some examples of these characteristics include change and generalized self-efficacy (Neves, 2009; Choi, 2011), dispositional resistance to change (Oreg et al., 2011), personal competence (Choi, 2011), locus of control (Holt et al., 2007), and positive affectivity (Oreg et al., 2011), among others. Although all these factors are important, Choi (2011) concluded in his review that individual-based factors have a lower importance in predicting change-related employee outcomes, especially compared to situational factors. Arising from the development of the context, content, and process areas of research, situational factors are indeed the aspects that have received most attention in the literature (Choi, 2011). The literature has shown the important role of numerous context-related factors in predicting change-readiness-related outcomes (Holt et al., 2007; Choi, 2011). For example, in terms of change-process factors, management processes allowing participation in the change project (Rafferty and Restubog, 2010), effective management–employee communication during the change process (e.g., Bordia et al., 2004), or the positive, successful change history of the individual in the organization (Devos et al., 2007) appear to have a positive influence. In terms of change content factors, the responses to change may become more positive insofar as the change is appropriate, favorable (Choi, 2011), and of less magnitude (Rafferty and Griffin, 2006). Finally, with regard to context-related factors, positive change-readiness outcomes can arise as a result of a supportive internal context (Armenakis and Bedeian, 1999), a clan-type culture where good human values such as loyalty, mutual trust, or friendship are dominant, and/or a leadership approach that creates quality leader–employee relationships and inspire trust (Choi, 2011).

Despite the important role of leadership in predicting employee outcomes (Hoch et al., 2016), the change-readiness literature has not yet dedicated the deserved space to this factor as an antecedent of employees’ level of readiness to change. Of the leadership approaches existing in the current literature, transformational leadership is the only one which has received attention (Choi, 2011); however, other more ethics-rooted forms of leadership (i.e., ethical leadership; Hoch et al., 2016) have received far less attention. These are much more connected to inspiring trust in employees (Hoch et al., 2016), and as a consequence, to driving positive change-readiness outcomes (Vakola, 2014). Thus, the role of ethical leadership in shaping employees’ readiness to change appears to be an intriguing area of research for learning more about how such an important outcome can be formed in organizations.

In studying the impact of ethical leadership in the workplace we cannot ignore the context in which this leadership is enacted. The important role of context in influencing leadership and its outcomes has been emphasized in the literature, recently (Porter and MacLaughlin, 2006; Osborn et al., 2014; Ng and Feldman, 2015; Oc, 2018). Oc (2018) notes that the context includes factors at the omnibus level such as *where*, *when*, and *who* is being led as well as factors at the discrete level such as *social*, *physical*, and *temporal* aspects. Of the omnibus level factors, the *where* dimension is likely one of the most studied (Oc, 2018), including the national culture. Hofstede (2010) argue that failure in implementing solutions at the organizational level is related to ignoring differences in the way leaders and followers think, feel, and act across different countries. Miroshnik (2002) also observed that the national culture and the social structures and values it embeds in people’s mindsets may influence the response of the employees to change. This is because the national culture may play an important role in shaping the personality; hence, influencing attitudes and behaviors (Hofstede, 2010) and likely changing the nature of the relationship between ethical leadership and its outcomes.

Considering Hofstede cultural framework (Hofstede Center, 1967–2010), Egypt is a country with features that could affect readiness to change levels as well as the ethical leadership relationship to employee readiness to change. For example, the preference for avoiding uncertainty and ambiguity in Egypt, the low score in long-term orientation – which leads to normative thinking and seeing change with suspicion – or the low score in indulgence – which indicates a high tendency to pessimism – (Hofstede Center, 1967–2010) should lead to low levels of readiness to change. The relatively high scores in power distance of this society, which fosters the use of centralized and autocratic management styles (Hofstede Center, 1967–2010), does not favor proactivity among employees either. In this context, however, the enactment of an ethical leadership approach is congruent with the collectivistic culture of this society (i.e., caring for others, Hofstede Center, 1967–2010) and could make a difference in fostering the levels of readiness to change among employees. As we will see below in detail, ethical leadership is congruent with some aspects (e.g., a better role clarification, promotion of trusting environments, Stouten et al., 2012) which could reduce Egyptians’ level of pessimism (De Hoogh and Den Hartog, 2008), change ambiguity avoidance, and fear to change. In addition, interactions with close, humane, empowering, and caring leaders such as ethical leaders (Kalshoven et al., 2011; Ng and Feldman, 2015) should be so pleasant in a society that expects the opposite (hierarchy-based and unequal leader–employee relationships) that a positive, proactive response such as readiness to change may emerge among employees with ease.

#### Ethical Leadership and Employees’ Readiness to Change

Researchers increasingly emphasize the organizational context as a major factor responsible for behavior at work. One significant organizational factor in this area is leadership, which is more effective if it is built on ethics and the welfare of followers (Melé, 2009). As such, it should come as no surprise that ethical leadership has attracted a high degree of research interest in recent years (Den Hartog, 2015; Bedi et al., 2016).

One of the most extended definitions of the term is that proposed by Brown et al. (2005). They defined ethical leadership as “the demonstration of normatively appropriate conduct through personal actions and interpersonal relationships and the promotion of such conduct to followers through two-way communication, reinforcement and decision-making” (Brown et al., 2005, p. 120). According to this definition, ethical leaders would serve as role models of ethical behavior who try to promote such a behavior in their followers, by using communication and reinforcement systems with which to communicate ethical standards and reward (discipline) ethical (unethical) behavior, respectively. Furthermore, although not explicitly noted in the definition, Brown et al. (2005) conceptualization implicitly involves avoiding harm onto the employees as well as acting in their best interests (Stouten et al., 2012).

Subsequently, De Hoogh and Den Hartog (2008) specified the concept by explaining the different behaviors an ethical leader usually undertakes. Specifically, De Hoogh and Den Hartog (2008) and Kalshoven et al. (2011, 2013) identified seven behavioral dimensions of ethical leadership: *fairness*, *power sharing*, *role clarification*, *ethical guidance*, *people orientation*, *concern for sustainability*, and *integrity*. First, ethical leaders are expected to *be fair* in their decisions, which entails being transparent, taking principled, balanced decisions (Kalshoven et al., 2011; Steinmann et al., 2016), being honest, acting responsibly, and treating employees equally (Kalshoven et al., 2013). Ethical leaders are also expected to *share power* (Kalshoven et al., 2011, 2013), which refers to allowing employees to participate in decision making, and listening to their ideas and opinions (Brown et al., 2005; De Hoogh and Den Hartog, 2008). Ethical leaders also *clarify roles*, by making performance goals, expectations, and responsibilities clear (De Hoogh and Den Hartog, 2008; Kalshoven et al., 2011). These leaders also show *ethical guidance*, as they communicate about ethics, explain ethical issues, and promote ethical conduct (Kalshoven et al., 2011, 2013). In particular, ethical leaders will do their best to make followers adopt ethical norms (Ng and Feldman, 2015). They are also *people-oriented*, by showing concern and care for people, and taking an interest in their welfare (Kalshoven et al., 2013). Further, they are *sensitive to environmental and sustainability issues*, which they demonstrate by caring about the impact of their actions on the society (Kalshoven et al., 2011, 2013). Finally, ethical leaders live with *integrity*, keeping their promises, acting consistently, and reflecting high word–deed alignment (Kalshoven et al., 2011).

With such behavioral features, it is no surprise that by exhibiting ethical leadership, managers have great potential to get the most from their relationship with their employees (Ruiz et al., 2011). Underlying all of these characteristics, however, is ethical behavior (Brown et al., 2005) and particularly, the leader’s true motivation to be ethical, which is relevant to understanding ethical leadership and its positive outcomes (Stouten et al., 2012). In Ng and Feldman’s words (2015), ethical leaders “uphold high ethical standards not only in their interactions with followers, but in virtually all aspects of their careers” (p. 950). This is critical to building leader’s sincerity and trustworthiness in the followers’ eyes (Stouten et al., 2012) and, in turn, to shape a high positive response among employees, including job dedication (Ng and Feldman, 2015). Thus, living an ethical life is key to understand ethical leadership effectiveness; it is by living this way how leaders are able to assure a peaceful environment and organize behavior in their small communities (Stouten et al., 2012).

Two theories help explain in detail how ethical leaders influence their employees. One is social exchange theory (Blau, 1964), which states that feelings of personal obligation, gratitude, and trust emerge in social exchanges. Drawing on the norm of reciprocity (Gouldner, 1960), this theory argues that when good treatment is received in social relationships, reciprocation will occur, possibly in the form of the exchange of goods of high value to the other party. In that connection, employees who perceive that managers are ethical and have their best interests at heart (Kalshoven et al., 2011, 2013) are likely to develop a greater dedication to the leader and the job (Ng and Feldman, 2015) and feel compelled to do something in return (Gouldner, 1960), such as making serious extra efforts (Brown et al., 2005). A second theory to explain the influence of ethical leaders getting the most from employees is social identity theory (Ashforth and Mael, 1989). According to this theory, if leaders are trustworthy, employees’ perceptions of oneness with the organization increases. Organizational identification also emerges if employees feel they are highly valued (Tyler, 1997). Thus, under situations where ethical leaders are consistent in word and deed, trustworthy, and people-oriented (Kalshoven et al., 2011, 2013), employees are more likely to do their best on behalf of their organizations.

One form of doing their best for the organization is to offer a positive response when the organization is undergoing change. When, in its drive to adapt to the marketplace, the organization implements change initiatives, it develops internal processes that break down existing structures and create new ones (Chonko, 2004). It is not surprising then that successful change management depends on employees’ acceptance and support of change (Abrell-Vogel and Rowold, 2014), which is a type of discretionary response (Herscovitch and Meyer, 2002) very proximal to readiness to change (Desplaces, 2005; Vakola, 2014). Readiness to change implies proactiveness and a positive attitude toward change (Vakola, 2014), and mental or physical preparedness to participate in any change (Desplaces, 2005), by performing actions that will improve, alter, vary, or modify something (Madsen et al., 2005). Such a positive response is more likely to be developed among employees under ethical leadership conditions that generate social exchange processes with employees, and inspire social identity in them. However, social exchange and social identity processes are not enough to bring about readiness to change in employees. Changes go from known to the unknown and challenge “the way things are done” (Vakola and Nikolaou, 2005), so high levels of uncertainty come as a result of times of change (Nelson et al., 1995). The negative emotions that change can bring to employees are numerous (e.g., anger, anxiety, chaos, depression, fear, etc.) (Vakola and Nikolaou, 2005), and ethical leadership can play an important role in mitigating all these negative emotions.

In effect, according to uncertainty reduction theory (Berger and Calabrese, 1975; Berger, 1986), individuals attempt to reduce uncertainty before acting. Employees make sense of the surrounding environment and events to obtain this uncertainty reduction (Neves et al., 2018). They seek clues that enable them to reduce uncertainty (Berger, 1986), and to trust the situation. Thus, observing ethical leadership in management can be helpful to achieve such a purpose, in line with Demerouti et al. (2001) job demands and resources (JD-R) theory. This theory posits that in facing job demands (i.e., aspects requiring physical, psychological efforts) job resources (i.e., aspects that help reduce job demands, and their corresponding physiological or psychological costs) can become a way to buffer the negative effects of job demands or stressors on the employees. Hence, under contexts of uncertainty (or job demands), the interaction with ethical leaders can be seen as a job resource that can help individuals to deal with this uncertainty and become more ready for change. In effect, ethical leaders, who are typically seen as representatives of the organization (Abrell-Vogel and Rowold, 2014), show integrity, take employees’ needs into consideration, provide a sense of confidence, and represent a valid source of ethical guidance (Kalshoven et al., 2011, 2013). In fact, under ethical leaders, employees will likely feel a supportive, fair, and humane treatment on a continuous basis and in the long run (Ng and Feldman, 2015), which should increase their security feelings. As a result, with ethical leaders, the uncertainty inherent to any organizational change might be reduced, with employees having their need for security met (Sharif and Scandura, 2014; Neves et al., 2018), and trusting their leaders (Sharif and Scandura, 2014), particularly their intention and behavior in the long run (Ng and Feldman, 2015) and the changes they initiate (Abrell-Vogel and Rowold, 2014). Under ethical leadership conditions, employees are more likely to feel less fear, more security (Sharif and Scandura, 2014; Neves et al., 2018), more information about the situation, and a greater sense of control (Morgan and Zeffane, 2003), which is key for them to offer their best response to any change process (Vakola and Nikolaou, 2005). Furthermore, ethical leaders empower people (Kalshoven et al., 2011, 2013), so they are more likely to promote employees’ involvement in the change process (i.e., voice in the change process; Sharif and Scandura, 2014), by reflecting confidence in employees’ abilities, thus increasing their self-efficacy perceptions (Abrell-Vogel and Rowold, 2014; Steinmann et al., 2016). Such enhancement in their self-efficacy perceptions is critical to understanding and responding to the environment in an efficient manner (Bandura, 2001) as well as to be ready for any change (Shah and Shah, 2010; Vakola, 2014).

Overall, social exchange theory (Blau, 1964), social identity theory (Ashforth and Mael, 1989), and uncertainty reduction theory (Berger and Calabrese, 1975) help to explain why ethical leadership, which encompasses positive attributes (e.g., integrity, fair and caring treatment of employees, and role clarification; Kalshoven et al., 2011), enhances employees’ readiness to change. Ethical leadership fosters quality social exchange relationships (social exchange theory; Walumbwa et al., 2011; Ng and Feldman, 2015) and perceptions of a sense of oneness with the leader and/or the unit or organization that the leader represents (social identity theory; Walumbwa et al., 2011). This is highly likely to stimulate in employees the efforts and positive attitudes needed to be ready for any change in the organization. Ethical leadership is also a source of role clarification and empowerment (Kalshoven et al., 2011), which can be useful in times of change to mitigate negative emotions and reduce uncertainty (uncertainty reduction theory; Neves et al., 2018) as well as to make employees feel more self-efficacious (Steinmann et al., 2016) and ready to respond to any change. Thus, the stronger development of social exchange, social identity, and uncertainty reduction processes that occur as a consequence of ethical leadership should result in a stronger positive influence of ethical leadership on employees’ readiness to change. Accordingly,

**H1**: Ethical leadership relates positively and directly to employees’ readiness to change.

### Ethical Leadership and Employees’ Readiness to Change: The Mediating Effect of an Organizational Culture of Effectiveness

Leaders play a critical role in shaping the work context (Politis, 2005; Neubert et al., 2009). Although they can influence the work environment by implementing formal systems, norms, and procedures, they do it mostly through their day-to-day informal behavior (Tenbrunsel et al., 2003). While Oc (2018), in his review on the role of context in understanding the leadership phenomenon emphasizes the role of context in shaping leadership and its outcomes, he also claims that this relationship can be reciprocal. The influence of leadership on the work context has been shown profusely (Zohar and Luria, 2005; Zohar and Tenne-Gazit, 2008; Neubert et al., 2009; Walumbwa et al., 2010; Zhang and Peterson, 2011), thus indicating the capacity of the leaders in influencing the way employees perceive their working environment (Porter and MacLaughlin, 2006; Oc, 2018). The aspects of this environment on which leaders can most influence include the organizational culture (Porter and MacLaughlin, 2006).

One of the leading authorities on organizational culture is Edgar Schein (1985, 1992), who defines the concept as “the pattern of shared basic assumptions that the group learned as it solved its problems of external adaptation and internal integration, that has worked well enough to be considered valid and, therefore, to be taught to new members as the correct way to perceive, think, and feel in relation to those problems” (Schein, 1992, p. 9). When we refer to the organizational level of analysis, these basic assumptions inject into the atmosphere values and beliefs on which individuals rely to guide their decisions and behaviors in an effective manner. However, the culture can put the emphasis on different, specific facets, or dimensions (e.g., safety culture, Mearns and Flin, 1999; ethical culture, Schaubroeck et al., 2012), and depending on the aspect it focuses on, the organizational culture will influence it in one way or another. One important facet to focus on is the general good functioning of the organization or its effectiveness. Therefore, if the organizational culture emphasizes this aspect, the organizational culture could be defined as the shared values and beliefs that individuals understand and consider as appropriate behavioral norms (Deshpande and Webster, 1989) in order to achieve organizational performance and effectiveness (Khuong and Nhu, 2015).

According to Sashkin (2012) and Sashkin and Rosenbach (2013), five key value dimensions foster the good functioning of organizations, and therefore make an organizational culture of effectiveness possible: *change management*, *goal achievement*, *coordinated teamwork*, *customer orientation*, and *shared values and beliefs*. With *change management*, organizational cultures are concerned about dealing with external forces and the need to adapt to change, thus making employees feel their destinies are a matter of internal control and enhancing their levels of self-confidence. By emphasizing *goal achievement*, organizational cultures of effectiveness also highlight the need to effectively achieve coherent and aligned goals, commonly leading to the empowerment of workers. Another important aspect emphasized in organizational cultures of effectiveness is *coordinated teamwork*, which highlights the importance of people working together to get the job done. Organizational cultures of effectiveness also emphasize *customer orientation*, which involves the need to continuously adapt to customers’ needs. Finally, the element which holds all these dimensions together is the *strength of shared values and beliefs.* It reflects the degree to which people agree that all the aforementioned values should guide their actions. Its relevance in building an organizational culture of effectiveness is clear (Sashkin, 2012, p. 4): “If everyone can buy into or reject them, at will, how can these values and beliefs have a consistent impact on people’s behavior?”

Based on this discussion of organizational culture of effectiveness and its components, an organization with *strong values and beliefs* regarding the importance of *adapting to change*, and working *to achieve specific goals* and targets, in *customer-oriented teams*, can enhance employees’ readiness to change. Choi’s (2011) review of the literature posits an organizational culture as an important antecedent of positive change-readiness responses. In particular, research has stressed that employees are more likely to be ready to change when the organizational culture emphasizes several aspects such as learning (Choi and Ruona, 2011), teamwork, collaboration (i.e., clan-type culture), energy, creativity, an emphasis on innovation (i.e., adhocracy-type culture), participation in decision making, or access to significant information in the workplace (Choi, 2011). It is of no surprise then that an organizational culture of effectiveness, which shares some of the cultural aspects described above, can positively influence employees’ readiness to change. In effect, by emphasizing the importance of *adapting to change*, employees are more likely to value the need and benefits of change (Weiner, 2009), and become more prepared for it (Armenakis and Harris, 2002). Such an organizational culture of effectiveness will result in positive attitudes and perceptions toward change (Self and Schraeder, 2009), which should increase employees’ confidence about their own abilities to cope with the situation, and thus increase their readiness to change (Vakola, 2014). Furthermore, by focusing on *goal achievement*, an organizational culture of effectiveness is more likely to empower employees and allow them to participate in the design and development of change, thus favoring less resistance to change (Reichers et al., 1997).

In addition, according to expectancy theory (Vroom, 1964), people choose courses of action based upon beliefs. Therefore, if these beliefs support the achievement of (aligned) goals, individuals will accept and be more willing to work on the achievement of goals for change, and will perform better during the change processes (Lines, 2004). The emphasis of an organizational culture of effectiveness on *fostering teamwork* also leads to greater readiness to change in employees. When employees interpret the team spirit positively, employees’ positive self-concepts are strengthened (Tajfel and Turner, 2004), which leads employees to be more predisposed toward change (Abrell-Vogel and Rowold, 2014). Eby et al. (2000), for example, found that individuals oriented toward working in teams appeared to be more receptive and ready to change. Finally, an organizational culture of effectiveness emphasizes *an orientation toward customers*, whose desires are highly changeable (Sashkin and Rosenbach, 2013). Thus, under a culture which emphasizes this aspect, employees are more likely to have more favorable attitudes toward change.

Although an organizational culture of effectiveness is defined as one important antecedent of employees’ readiness to change, leaders play a critical role in its development and construction (Schein, 1992). In Schein’s (1992) view, leaders influence the below-the-surface (values and beliefs), but also the surface layers of an organization’s culture, including visible artifacts such as behavioral norms, policies, and standards (Schein, 2010). Therefore, the values that leaders hold are critical to configuring the emphasis of the organizational culture; if these values are ethically rooted, then an organizational culture of effectiveness is highly likely (cf., De Hoogh and Den Hartog, 2008; Hu and Liden, 2011). In fact, ethical leadership and an organizational culture of effectiveness both place a strong emphasis on certain aspects that help organizations gain in terms of effectiveness. For example, for ethical leaders, stepping outside oneself and focusing on other stakeholders’ interests are important (Kalshoven et al., 2011, 2013). Therefore, organizations led by ethical leaders are more likely to be aware of stakeholders’ concerns (De Hoogh and Den Hartog, 2008), and to be more oriented to meet customers’ needs (Lindblom et al., 2015).

In addition, ethical leaders empower and develop employees, and provide the information needed to complete tasks (Kalshoven et al., 2011, 2013), which should make it easier for employees to learn, and apply new skills and new technologies to achieve organizational goals (Ozaralli, 2003). Ethical leaders also incorporate employees’ ideas into their decisions, involving them in goal setting (Kalshoven et al., 2011; Steinmann et al., 2016). As a result, goals are better clarified and employees perceive them as their own, which should motivate greater efforts to achieve them (Loceke and Latham, 1990). Interestingly, with ethical leaders, cooperativeness and a sense of trust in others is likely to emerge (Den Hartog and De Hoogh, 2009), which facilitates teamwork and brings people’s efforts together to achieve organizational goals (Sashkin, 2012; Sashkin and Rosenbach, 2013). Finally, since employees tend to be attracted to ethical environments (e.g., Ruiz-Palomino and Martínez-Cañas, 2013), cultures shaped by ethical leadership should, consequently, be more strongly shared by employees, which should enhance organizational effectiveness (Sashkin, 2012; Sashkin and Rosenbach, 2013).

Overall, this reasoning leads us to suggest that ethical leadership may positively influence employees’ readiness to change, through shaping an organizational culture of effectiveness. Although, as argued in H1, ethical leadership is expected to directly and positively influence employees’ readiness to change, based upon social exchange, social identity, and uncertainty reduction reasoning, an organizational culture of effectiveness is also likely to help in explaining this relationship. It could be the mechanism that helps to complete the social exchange, social identity, and uncertainty reduction explanations for the “ethical leadership–employees readiness to change” relationship. In effect, ethical leadership, by exhibiting integrity, fairness, and genuine concern for employees (Kalshoven et al., 2011), is more likely to invoke gratitude in employees, leading to social exchange relationships (Blau, 1964) in which positive employee responses such as readiness to change can easily arise. Due to the strong ethical standards that ethical leadership encompasses – which makes it easier for employees to feel they are highly valued-ethical leadership stimulates self-identification processes with the leader and organization, which lead to employees making extra efforts for the organization (Walumbwa et al., 2011), including a greater readiness to change. Finally, according to uncertainty reduction theory, ethical leadership, through serving as a strong guide in terms of values (Kalshoven et al., 2011), would serve to mitigate the negative emotions that any change entails and would help stimulate positive change-readiness-related responses by employees.

While social exchange theory and social identity theory help explain the positive influence of ethical leadership on employees’ readiness to change via stimulating a positive response in the employees, these mechanisms lack the importance that the transmission of a set of values that shape readiness to change may also have. Uncertainty reduction theory, the remaining mechanism used in H1 to explain the ethical leadership–employee readiness to change relationship, includes, to some extent, the important role of setting values in the organization to reduce ambiguity and uncertainty, but ignores the important role of shaping values that drive employees toward making positive readiness-to-change-related responses themselves. An organizational culture of effectiveness, however, would enable this idea to be included and would therefore complete the social exchange, social identity, and uncertainty reduction explanations regarding why ethical leadership may positively relate to employees’ readiness to change.

The underlying set of values of any organizational culture shapes the behavior of the employees in the same direction; employees importantly rely on the values espoused in the organizational culture to guide their behaviors (Schein, 1985, 1992). As such, under organizational culture of effectiveness conditions, and therefore under conditions where the organizational culture emphasizes a set of values that are conducive to positive responses concerning organizational change, employees’ readiness to change is more likely. Thus, organizational culture of effectiveness becomes an important mechanism by which ethical leadership, of both upper– and middle–lower echelons, is likely to influence employees’ readiness to change. In effect, while upper echelons exert a significant influence on the content of the organizational policies and practices, and therefore in the set of values that are required to be spread in the organization, middle–lower echelons play an important role in the extent to which employees internalize these values (Steffensen et al., 2019). Managers in middle–lower echelons create more meaningful relationships with their employees, and their support regarding the set of values taught as the correct way to think and feel in the organization (i.e., organizational culture) turns them into strong influences of the employees’ acceptance of and commitment to such a set of values (Steffensen et al., 2019). Furthermore, in line with Schaubroeck et al. (2012) findings about the important role of middle–lower echelons in shaping the organizational ethical culture, it is of no surprise that these managers can play an important role in shaping an organizational culture of effectiveness.

In summary, although ethical leadership is likely to influence employees’ readiness to change for social exchange (Blau, 1964), social identity (Ashforth and Mael, 1989), and uncertainty reduction (Berger and Calabrese, 1975) processes, its ability to shape an organizational culture of effectiveness makes it likely that an organizational culture of effectiveness completes this relationship. An organizational culture of effectiveness would thus partially mediate the positive relationship of ethical leadership to employees’ readiness to change. This mediation would be partial because the interaction with ethical leaders itself is enough to make employees want to reciprocate with valuable behavior – social exchange theory, Blau (1964) – feel identified with their organization – social identity theory, Ashforth and Mael (1989) – or experience less uncertainty under any change process – uncertainty reduction theory, Berger and Calabrese (1975) – which is consistent with attitudinal and behavioral processes leading to a higher readiness to change. Accordingly,

**H2**: An organizational culture of effectiveness partially mediates the relationship between ethical leadership and employees’ readiness to change.

## Materials and Methods

### Sample and Procedure

To test the hypotheses, three of the largest public foreign trade organizations in Egypt were considered. After obtaining the approval of their general managers, questionnaires were randomly distributed to a sample of 357 employees who directly reported to middle and lower-level managers in these three organizations. The sample size was calculated by randomly selecting one employee for each leader and following recommendations regarding the amount of data to collect on each organization to obtain a representative sample (Cochran, 1977). Participation was voluntary and the data were collected in two rounds. In the first round, employees assessed the ethical leadership of their direct managers and the extent to which an organizational culture of effectiveness was present in the organization. A second round, 4 weeks later, measured their own readiness to change using employees’ own scores. Data were collected in Arabic to guarantee an accurate interpretation of all questions. Scales were, consequently, translated into the Arabic language prior to data collection using the back-translation method (Brislin, 1980), and this method revealed no significant differences between the English and Arabic versions of the scales. For the purposes of the current investigation, data from temporary employees who had been working for <1 year with their current organizations were excluded. In the end, 270 usable surveys were obtained, with a response rate of 75.63%, which is quite high considering the sensitivity of the ethical content of the research (Randall and Gibson, 1990) and given that various departments were surveyed (Valentine et al., 2006). Table 1 shows the demographics of the study sample.

**TABLE 1:** Sample characteristics (*N* = 270).

	**Frequency**	**% of total**		**Frequency**	**% of total**
**Age**			**Years of experience in the job**		
25–34 years old	39	14.40			
35–44 years old	120	44.40	<5 years	27	10.00
45–54 years old	85	31.50	5–10 years	61	22.60
>55 years old	26	9.60	>10 years	182	67.40
**Gender**					
Male	173	64.10			
Female	97	35.90			
**Level of education**			**Job type**		
Secondary studies	94	34.80	Non-supervision role	89	33.00
Bachelor’s degree	160	59.30	Supervision role	181	67.00
Master’s degree	16	5.90			

To reduce the threat of common method variance, evaluation apprehension, and social desirability bias, the questionnaire design followed several salient recommendations (Podsakoff et al., 2003). A cover letter emphasized that there were no right or wrong answers. Although each questionnaire included an identification code, anonymity and absolute confidentiality were guaranteed (respondents did not have to reveal their names, their jobs, or the organizations they worked for). Finally, two steps specifically sought to mitigate common method bias: (1) temporal separation in the questionnaire between predictors (ethical leadership, organizational culture of effectiveness) and criterion variables (employees’ readiness to change); and (2) survey items that were simple, specific, and concise, according to the pilot test results.

### Measures

All variables in this study used a five-point Likert response format (1 = “strongly disagree,” 7 = “strongly agree”). All of the items used appear in the Supplementary Appendix.

#### Ethical Leadership

Kalshoven et al. (2011) psychometrically robust 38-item scale was used to measure the ethical leadership of middle–lower managers, specifically the various ethical leader behaviors that comprise this scale, namely, people orientation (seven items), power sharing (six items), fairness (six items), concern for sustainability (three items), ethical guidance (seven items), role clarification (five items), and integrity (four items). This scale was used here over other options (i.e., Brown et al., 2005) because it identifies the totality of the dimensions that form this phenomenon properly (Steinmann et al., 2016). Furthermore, this scale is suitable in this study for noting specific behavioral dimensions that favor readiness to change among employees. For example, its *role clarification* and *power sharing* behavioral dimensions are suggested to help employees to feel they can count on the information they may need at any time and they are self-efficacious, respectively (Steinmann et al., 2016).

In line with Kalshoven et al. (2011, 2013), and after reverse items (see theSupplementary Appendix) had been reverse scored properly, these behaviors were combined into an overall score. Due to low loadings, nine items were dropped: two from people orientation (“sympathizes with me when I have problems,” “cares about his/her followers”), two from fairness (“holds me responsible for things that are not my fault, manipulates subordinates”), one from concern for sustainability (“stimulates recycling of items and materials in our department”), two from ethical guidance (“clearly explains integrity-related codes of conduct,” “clarifies the likely consequences of possible unethical behavior by myself and my colleagues”), one from role clarification (“explains what is expected of me and my colleagues”), and one from integrity (“can be relied on to honor his/her commitments”). The scale used a Likert response format (1 = “strongly disagree,” 5 = “strongly agree”). The responses were averaged for each respondent such that higher scores indicated a stronger ethical leadership. Sample items used are “can be trusted to do the thing(s) he/she say(s) he will do” and “shows concern for sustainability issues.” The overall ethical leadership scale for the remaining 29 items (9 items were dropped because of low loadings) had a Cronbach’s alpha of 0.90.

#### Organizational Culture of Effectiveness

To assess organizational culture of effectiveness, a selection of items from Sashkin and Rosenbach’s (2013) scale based on Sashkin’s (2012) previous research was used. This scale measures the different aspects that an organizational culture of effectiveness must include to promote organizational effectiveness (i.e., change management, goal achievement, coordinated teamwork, shared values and beliefs, and customer orientation). Our choice of this scale rested upon the high potential of most of these aspects to favor an environment which is supportive of readiness to change. In effect, an emphasis on customer orientation and change management is likely to favor that employees become more adapted to customer needs (Franke and Park, 2006) or any organizational change that can occur (Armenakis and Harris, 2002). In addition, by emphasizing goal achievement, achievement orientation is favored, so willingness to learn new skills and become prepared for any emerging change may arise (Caldwell et al., 2009). Finally, when teamwork is encouraged, employees may feel they have enough support to cope and adapt to any change (Choi, 2011).

In this study, three items from each dimension were selected for measuring this variable, and respondents had to indicate the extent to which each one was present in their organizations. The scale used a Likert response format (1 = “strongly disagree,” 5 = “strongly agree”), and the responses for each respondent were averaged. Because some items were reverse worded (see the Supplementary Appendix), we reverse coded these items, so that the direction of the revealed relationships could reflect the wording of the hypotheses. Thus, higher scores indicated a stronger organizational culture of effectiveness. The scale’s alpha reliability was 0.88.

#### Employee Readiness to Change

Drawing on the *Change Readiness Survey* (WorkLife Design, 2008), a selection of three adapted items was used. While other scales are available (Hanpachern et al., 1998; Madsen et al., 2005), we used this scale, and in particular, three items, because these reflected the motivation and attitudes of the participants to engage with the change and make it work, thus capturing the *readiness to change* conception properly. In addition, the instructions this survey provides to participants before responding the items allow them to put themselves in context, which can help to gauge this variable in a reliable manner. In particular, participants were asked to think about how their current organization typically plans for and implements workplace changes and with this “change history” in mind, they were asked to indicate their level of agreement about how they had faced such changes in the past, using a Likert response format (1 = “strongly disagree,” 5 = “strongly agree”). The responses for each respondent were averaged such that higher scores indicated a stronger employee readiness to change. A sample item was “when I am affected by organizational change, I am involved in identifying possible obstacles.” The scale’s alpha reliability was 0.72.

#### Control Variables

The results were controlled for age, tenure in the job, gender, and education, all of which can potentially relate to organizational behavior, and specifically to employees’ readiness to change (Shah and Shah, 2010). For example, although findings are mixed regarding age (Kunze et al., 2013), this demographic factor is suggested to affect readiness to change negatively; older employees are associated with stability and a lower potential for learning new skills (Finkelstein et al., 1995), which should lead them to a stronger psychological inability to accepting radical change (Dunks, 2000). As with age, tenure in the job is expected to affect readiness to change negatively; with increasing tenure in the job, the employees will show little contact with other work situations, therefore increasing their levels of cognitive rigidity and aversion to change (Kunze et al., 2013). Regarding the effect of gender a number of studies show no relationship (Cunningham et al., 2002; Madsen et al., 2005); however, females are seen as more risk-averse (Byrnes et al., 1999), and more apprehensive toward any change (Collins, 2005), which should lead to lower levels of readiness to change (Dhingra and Punia, 2016). Finally, because the level of education is linked to seeing the change as something which is necessary and beneficial (Alas, 2009), this variable should relate positively to readiness to change, in line with previous findings (Samaranayake and Takemura, 2017; Arnéguy et al., 2018). While age and education mimicked continuous variables, a dummy coded variable was created for gender (0 = male, 1 = female). For age and level of education, an interval scale anchored at 1 (younger/lower educated employees) and 5 (older/higher educated employees) was used; for years of experience in the job, the interval scale ranged between 1 (<5 years) and 3 (>10 years).

### Data Analysis

SPSS 24.0 was used to (1) obtain descriptive statistics and (2) run an exploratory factor analysis to examine the potential for common method variance in the data (Podsakoff et al., 2003). Then, for the testing of hypotheses, PROCESS (Hayes, 2013) was used. The hypotheses were tested through running bias-corrected bootstrap analyses at a 99% level of significance (using 5,000 subsamples) via Hayes’ PROCESS macros with PROCESS v2.10 (Hayes, 2013). While bootstrapping treats the original sample as the population, this method resamples (with replacement) observations from within that sample thousands of times over to generate sample-based estimates of the population values (Hayes, 2013). This method is suitable for mediation as it helps to estimate indirect effects, confidence intervals, and standard error (Hayes, 2013).

## Analysis and Results

### Common Method Variance

To check for common method variance, and thus to assess whether variance in the data could be attributed to a single factor, the Harman’s one-factor test was run. This test, which involves an exploratory factor analysis of the data, revealed an unrotated factor solution involving 12 factors with eigenvalues >1. Because the first factor explained less than half (36%) of the total variance (78%), common method variance does not appear to affect the study findings (Podsakoff et al., 2003).

We also ran Lindell and Whitney’s (2001) marker variable technique to confirm these results. In essence, the marker variable technique uses a (marker) variable that is not theoretically related to any substantive variable of the study to calculate common method variance, and thus adjust the correlations among the study constructs. Although demographic variables are found not to be the most desirable option, there are many examples in the literature that use this option as a marker variable (cf., Williams et al., 2010; Simmering et al., 2014). Thus, in running the marker variable technique, we used the respondent’s job type (0 = non-supervision role; 1 = supervision role) as a variable that was theoretically unrelated to any substantive study variable and could meet the necessary conditions to be a marker variable. As expected, this marker variable was not significantly correlated with any of the study variables (Table 2). Furthermore, following Lindell and Whitney’s (2001) recommendations, the lowest absolute correlation between the marker variable and the substantive study variables (rm = 0.05) was partialled out from the uncorrected correlations to check for the magnitude and significance of common method variance. After controlling for common method variance, all correlations that were previously significant remained significant, so we can conclude that common method variance is unlikely to have affected our findings in the current study.

**TABLE 2:** Descriptive statistics and correlation matrix (*N* = 270).

**Descriptive statistics**			**Correlation matrix. Cronbach’s alphas in bold (in the diagonal)**							
	**Mean**	***SD***	**1**	**2**	**3**	**4**	**5**	**6**	**7**	**8**
1. Ethical leadership	3.61	0.52	**0.90**							
2. OCE	3.49	0.61	0.86^∗∗^	**0.88**						
3. Years of experience in the job	2.57	0.67	–0.29^∗∗^	–0.28^∗∗^	n.a.					
4. Gender	n.a.	n.a.	0.12^∗^	0.17^∗∗^	0.05	n.a.				
5. Age	2.36	0.84	–0.00	0.11	0.38^∗∗^	–0.06	n.a.			
6. Level of education	1.71	0.57	–0.28^∗∗^	–0.28^∗∗^	0.15^∗^	–0.08	–0.04	n.a.		
7. Job type	n.a.	n.a.	–0.06	–0.10	–0.02	–0.06	0.26^∗∗^	–0.32^∗∗^	n.a.	
8. Employee readiness to change	3.32	0.83	0.74^∗∗^	0.77^∗∗^	–0.22^∗∗^	0.13^∗^	0.03	–0.18^∗∗^	–0.05	**0.72**

*^∗∗^*p* < 0.01; ^∗^*p* < 0.05 (two-tailed test). *SD*, standard deviation; n.a., not applicable; OCE, organizational culture of effectiveness. Gender and job type were coded as dummy variables (1 = male, 2 = female; 0 = supervision role; 1 = non-supervision role), so mean and SD were not applicable; percentages were instead calculated, with values of 64.1% for males and 67% for supervision roles. Interval scales were used for measuring years of experience in the job (1 ≤ 5 years, 2 = between 5 and 10 years, 3 ≥ 10 years), age (1 = up to 24 years old, 2 = between 25 and 34 years old; 3 = between 35 and 44 years old; 4 = between 45 and 54 years old; 5 = 55 or more than 55 years old), and level of education (1 = primary studies; 2 = secondary studies; 3 = bachelor’s degree; 4 = master’s degree; 5 = Ph.D./doctorate).*

### Preliminary Analysis

Means, standard deviations, and inter-correlations for both study and control variables are presented in Table 2. Table 2 also provides the reliability levels of the measures utilized, with all the alpha reliabilities for the scales far above the 0.70 threshold (Hair et al., 2006). Prior to conducting the analyses to test the hypotheses, we checked the need to include control variables according to recent recommendations (Bernerth and Aguinis, 2015). First, the statistical analysis was conducted with all control variables. Next, the analysis included only the control variables that were significantly correlated with the mediator and/or the dependent variable (i.e., age was not related significantly and was excluded). Finally, the analysis was performed without including any of the control variables. The comparison of results of these three analyses revealed identical parameters; furthermore, the significance levels and confidence intervals remained unchanged. Thus, in line with Bernerth and Aguinis (2015) we decided to present our results concerning the hypothesis testing without the effects of control variables.

### Hypothesis Testing

Concerning the hypotheses, Table 3 shows empirical support for both H1 and H2. In support of H1, the findings confirm the predictions. Table 3 reveals that ethical leadership is positively and directly related to employees’ readiness to change. Even when the mediator is included in the model, the direct effect between ethical leadership and employees’ readiness to change is significant and positive (unstandardized beta = 0.47, standard error = 0.12, *p* < 0.001), thus leading us to accept H1.

**TABLE 3:** Regression results with PROCESS (*N* = 270).

	**Organizational culture of effectiveness (*R*^2^ = 0.74)**				**Employee readiness to change (*R*^2^ = 0.61)**		
**Variable**	***B***	***SE***	***t***		***B***	***SE***	***t***
Constant	−0.16	0.13	−1.24		−0.97^∗∗^	0.33	−2.88
Organizational culture of effectivenes					0.71^∗∗∗^	0.10	6.57
Ethical leadership	1.00^∗∗∗^	0.03	28.15		0.47^∗∗∗^	0.12	3.84

	**Bootstrapping effect**			***SE***	**99% BCA CI (LL, UL)**		

Indirect effect of ethical leadership on employee readiness to change (via organizational culture of effectiveness)		0.72		0.15	0.38	1.20	

*Unstandardized regression coefficients; bootstrap sample size = 5,000; 99% BCA CI, bias-corrected and accelerated confidence interval; LL, lower limit; UL, upper limit; SE, standard error. ^∗∗^*p* < 0.01. ^∗∗∗^*p* < 0.001.*

Regarding the partial mediation hypothesis, an examination of Baron and Kenny’s (1986) four conditions provides initial support for H2. First, the predictor, ethical leadership, is significantly associated with the mediator, organizational culture of effectiveness (unstandardized beta = 1.00, standard error = 0.03, *p* < 0.001; Table 3). Second, the predictor is associated positively and significantly with the dependent variable, employees’ readiness to change (unstandardized beta = 1.15, standard error = 0.07, *p* < 0.001; Table 4). Third, the mediator is significantly associated with the dependent variable (unstandardized beta = 0.71, standard error = 0.10, *p* < 0.001; Table 3). Finally, the effect size of the predictor, ethical leadership, on the dependent measure, employees’ readiness to change, is lower after controlling for the mediator, organizational culture of effectiveness, but remains significant in support of a partial mediation effect (unstandardized beta without the mediator = 1.15, standard error = 0.07, *p* < 0.001; unstandardized beta with the mediator = 0.47, standard error = 0.12, *p* < 0.001; Table 4). Thus, the fulfillment of all these conditions provides initial support for H2.

**TABLE 4:** Ethical leadership and employee readiness to change: direct versus mediated effect model.

	**Variance explained**			**Mediation strength**
	Direct model	Mediated model	Δ Variance explained	(*f*^2^) Effect size
Employee readiness to change	0.55	0.61	0.06	0.15 (moderate to large)

	**Unstandardized beta (*SE*)**			**Kappa squared**
	
	Direct model	Mediated model		Indirect effect = 0.32 (large)
Employee readiness to change	1.15^∗∗∗^ (0.07)	0.47^∗∗∗^ (0.12)		

**f*^2^ = (*R*^2^ included − *R*^2^ excluded)/(1 − *R*^2^ included). Effect sizes of *f*^2^ between 0.15 and 0.35 are moderate to large in size (Cohen, 1988). Kappa-squared effects higher than 0.25 are large (Cohen, 1988; Preacher and Kelley, 2011). ^∗∗∗^*p* < 0.001. SE, standard error.*

For mediation to exist, however, the indirect effect between these variables must be significant (Hayes, 2013). The 99% bias-corrected and accelerated percentile bootstrap method with 5,000 repetitions revealed a significant indirect effect of ethical leadership on employees’ readiness to change through organizational culture of effectiveness (indirect effect = 0.72, standard error = 0.15, 99% bias-corrected and accelerated confidence interval = 0.38, 1.20; Table 3); the Sobel test for this indirect effect confirmed the existence of mediation (indirect effect = 0.79, standard error = 0.11, *z* = 6.40, *p* < 0.001). Thus, the positive impact of ethical leadership on employees’ readiness to change is partially mediated by organizational culture of effectiveness, in support of H2. With the mediator, the variance explained in employees’ readiness to change increases from 0.55 to 0.61 (Δ*R*^2^ = 0.06), thus implying a moderate to large mediating effect of organizational culture of effectiveness between ethical leadership and employees’ readiness to change (*f*^2^ = 0.15; Cohen, 1988; Table 4). Preacher and Kelley’s (2011) Kappa-squared test confirmed the importance of this mediation effect size. This test revealed an indirect effect which is about 32% of its possible maximum value, which, in accordance to recommended guidelines (*R*^2^ effects of 0.01, 0.09, and 0.25 indicating small, medium, and large effects, respectively; Cohen, 1988), is identified as large in size (indirect effect = 0.32, standard error = 0.05; 99% bias-corrected and accelerated confidence interval = 0.21, 0.43; Table 4).

## Discussion and Conclusion

### Theoretical Contributions

The organizational change literature suggests that leadership may play an important role in influencing employees’ attitudes and behavior toward change (Abrell-Vogel and Rowold, 2014). Recent research has also revealed positive behavioral outcomes of ethical leaders in change conditions (Sharif and Scandura, 2014). However, until now no study had examined the direct effects of ethical leadership on employees’ readiness to change, or had investigated the mediating mechanisms that could explain this relationship.

The aim of this investigation was to analyze whether ethical leadership positively influences employees’ readiness to change and whether an organizational culture of effectiveness mediates this relationship. The findings support these hypotheses. The results indicate that ethical leadership has significant, direct positive effects on employees’ readiness to change. The results also reveal that an organizational culture of effectiveness mediates this relationship, thus indicating that ethical leadership shapes cultural elements that prompt organizational effectiveness, and that by shaping such an organizational culture, ethical leadership helps to enhance employees’ readiness to change. Overall, this study contributes novel theoretical implications to the literature on leadership, organizational culture, and organizational change management.

This research accordingly responds to recent calls to investigate the role of ethical leadership in organizational change situations (i.e., Sharif and Scandura, 2014), and extends previous findings indicating greater employee readiness to change in contexts where trust in management, management support, and good management–employee relationships abound (Shah and Shah, 2010; Shah, 2014; Vakola, 2014; Kirrane et al., 2017). The investigation also advances previous literature suggesting the positive links of ethical leadership-related approaches to organizational effectiveness (i.e., ethical leadership, De Hoogh and Den Hartog, 2008; servant leadership, Hu and Liden, 2011) by indicating that ethical leaders shape cultural elements leading to organizational effectiveness (i.e., change management, goal achievement, coordinated teamwork, customer orientation, shared values and beliefs strength; Sashkin, 2012; Sashkin and Rosenbach, 2013). However, although such cultural aspects may explain the positive relationship between ethical leadership and employees’ readiness to change, the findings reveal that such mediation is marginal, and reveal the important role of ethical leadership in accounting for this valuable employee outcome itself.

Overall, this study brings ethical leadership, the organizational culture of effectiveness, and employees’ readiness to change together for the first time in the literature, and does so in a non-Western context (i.e., Egypt). This is important in the context of the current ethical leadership literature, which over-focuses on Western contexts. This study is set in an Arab culture and reveals the influence of ethical leadership on one type of job response that is positive for the organization, namely, readiness to change, thus helping generalize existing theories across different contexts (Whetten, 2009).

### Managerial Implications

The findings of this study reveal some practical ideas for managing organizational change. First, organizations interested in succeeding when coping with change should put ethical leaders in management positions. The more ethical leaders there are in such positions, the easier it is for employees to participate in decisions about the organization’s future. Therefore, feelings of insecurity can be reduced (cf., Ng and Feldman, 2015), and trust/control over the situation can be increased, which should help employees be more self-confident, and thus more ready to change. In trying to standardize ethical leadership across the organization, hiring processes should emphasize ethical leadership traits. These processes, in combination with other useful practices to shape ethical leadership at management levels (e.g., performance assessments, training, role modeling, rewards systems), should help managers to develop ethical leadership behaviors (e.g., fair decisions or behaviors, empowering behavior, people-oriented behavior, ethical-guidance behavior, role clarification, concern for sustainability, and integrity) in their daily activities.

However, because only one bad, specific behavior is enough to damage the reputation of ethical leadership, human resource managers should make great efforts to design training programs oriented to managers developing these ethical leadership behaviors in an automatic and natural way. According to Ajzen and Fishbein’s (1980) theory of reasoned action, the desirability of the behavior and the subjective norms (social pressure to develop such behavior) are critical antecedents of behavioral intentions. As such, training initiatives should first aim for managers to internally desire such behavior and become aware of how important doing so is for organizational members, especially when the organization is undergoing changes. In addition, such training programs should serve to enhance the cognitive-autonomous reasoning of managers to connect them to their sense of self (Rozuel and Kakabadse, 2010), with which to incorporate such ethical behaviors cognitively, and make it easier for managers to develop these behaviors in an automatic and natural way (Aarts et al., 1998).

Second, if it is not possible to develop ethical leadership, managers have other means to motivate employees’ readiness to change. For example, they might shape an organizational culture of effectiveness that emphasizes aspects which, when strongly shared by employees, may facilitate readiness to change in the workplace. In particular, managers can emphasize aspects such as: “we are able to affect the environment and manage the change,” “we need to achieve goals and improve, constantly,” “we need to work as a coordinated team,” and “we need to meet our customers’ needs.” These aspects are critical to gain organizational effectiveness, as they all help build a workforce that is eager to improve, and confident enough about their resources (i.e., team colleagues) and abilities to face new challenges. It makes these people more open to change (Kobasa, 1982), and thus more likely to give support to change and engage in change efforts. However, managers should realize that the best way to shape such an organizational culture of effectiveness is by endeavoring to develop ethical leadership behaviors on a daily basis, and to make these behaviors emerge in an automatic, natural way, thus becoming habits.

### Limitations and Future Research Directions

As with any field research, the present research contains several limitations that offer opportunities for further research efforts. First, this study was conducted in a specific industry and cultural context (i.e., public foreign trade in Egypt), so the generalization of the findings of this study demands caution. These findings are in line with existing theories, but Egyptian culture presents contextual characteristics that may have affected them. For example, Egypt is highly collectivistic (Hofstede Center, 1967–2010), so people closely identify with the group or team to which they belong, which could have contributed to the mediating effect of an organizational culture of effectiveness that emphasizes teamwork. In addition, Egypt is highly avoidant of uncertainty, short-term oriented – which leads to see the change with fear – and restrained (Hofstede Center, 1967–2010). Accordingly, the participants in this study could have been more sensitive to the influence of ethical leadership, as it is a factor that may reduce the level of uncertainty (Sharif and Scandura, 2014), fear (Sharif and Scandura, 2014; Neves et al., 2018), and pessimism (De Hoogh and Den Hartog, 2008) among employees. Thus, further research should include additional industries and design cross-cultural studies to test for the universality (or context sensitivity) of the findings of this study and their underlying theories.

Second, the data were cross-sectional, which limits the causal conclusions. However, prior meta-analytic research suggests that ethical leadership is a strong antecedent of positive outcomes in employees (Bedi et al., 2016), which suggests that causality leads from ethical leadership to employees’ readiness to change, not the reverse. However, future work might address longitudinal studies to provide stronger evidence of the causal relations herein identified.

Another limitation is that the study variables were based on self-reported data, which can create common method bias concerns. The current study followed procedural remedies to avoid this bias (Podsakoff et al., 2003) and the *post hoc* test that was conducted suggests that this bias was not a problem. However, future research could extend the findings by measuring employees’ readiness to change using team colleagues’ or managers’ scores.

Finally, this investigation focused on managers’ ethical leadership as a trigger for an organizational culture of effectiveness and employees’ readiness to change. However, interactions with team colleagues who have a strong ethical focus might also play a role here. Shah and Shah (2010), for example, find that having good relations with peers, who play a role in shaping the organizational culture (Harris, 1994), increases employees’ readiness to change. Thus, future studies could evaluate this possibility in accounting for employees’ readiness to change. Overall, the findings of this study provide interesting insights for management literature and offer new opportunities for future research.

## Ethics Statement

Ethics approval was not required for this study, as per the guidelines of the University of Deusto. Participation was voluntary, and the purpose of the research was clearly explained in a cover letter. Informed and written consent was obtained from all participants. A group discussion session was conducted with the first author prior to questionnaire distribution where informed consent was obtained. Benefits and objectives of the study were explained in that session and participants were assured that their participation would be voluntary. They were then asked to sign the consent form and proceed to the questionnaire if they accepted taking part in the study. Participants were guaranteed that their data would be used for academic purposes only and that their responses would never be shared with their leaders. The purpose and procedures of the study were carefully illustrated to all participants and they were free to withdraw at any time.

## Author Contributions

MM and DM conceived the presented idea, worked initially on the theory, and performed the computations. PR-P and MM developed and added value to the theoretical framework, and conducted the review of the study with LG. LG verified the analytical methods, supervised the findings, and contributed to the design of the study.

## Conflict of Interest

The authors declare that the research was conducted in the absence of any commercial or financial relationships that could be construed as a potential conflict of interest.
