# Supplementary material for: How Ethical Leadership Shapes Employees’ Readiness to Change: The Mediating Role of an Organizational Culture of Effectiveness
Source: Front Psychol. 2019 Nov 14;10:2493. doi: 10.3389/fpsyg.2019.02493 (PMC6874171; doi:10.3389/fpsyg.2019.02493)
Supplement: Supplementary file 1 [file Data_Sheet_1.docx]

**Appendix. Measures**

**Ethical Leadership** (Kalshoven et al., 2011).

**___***People orientation*

Is interested in how I feel and how I am doing

Takes time for personal contact.

Pays attention to my personal needs.

Takes time to talk about work-related emotions.

Is genuinely concerned about my personal development

*Sympathizes with me when I have problems (dropped)*

*Cares about his/her followers (dropped)*

**___***Fairness*

Holds me accountable for problems over which I have no control* (reverse item)

Holds me responsible for work that I gave no control over* (reverse item)

*Holds me responsible for things that are not my fault (dropped)* (reverse item)*

Pursues his/her own success at the expense of others* (reverse item)

Is focused mainly on reaching his/her own goals* (reverse item)

*Manipulates subordinates (dropped)* (reverse item)*

**___***Power sharing*

Allows subordinates to influence critical decisions

Does not allow others to participate in decision making* (reverse item)

Seeks advice from subordinates concerning organizational strategy

Will reconsider decisions on the basis of recommendations by those who report to him/her

Delegates challenging responsibilities to subordinates

Permits me to play a key role in setting my own performance goals

**___***Concern for Sustainability*

Would like to work in an environmentally friendly manner

Shows concern for sustainability issues

*Stimulates recycling of items and materials in our department (dropped)*

**___***Ethical guidance*

*Clearly explains integrity related codes of conduct* *(dropped)*

Explains what is expected from employees in terms of behaving with integrity

Clarifies integrity guidelines

Ensures that employees follow codes of integrity

*Clarifies the likely consequences of possible unethical behavior by myself and my colleagues (dropped)*

Stimulates the discussion of integrity issues among employees

Compliments employees who behave according to the integrity guidelines

**___***Role clarification*

Indicates what the performance expectations of each group member are.

Explains what is expected of each group member

*Explains what is expected of me and my colleagues (dropped)*

Clarifies priorities

Clarifies who is responsible for what

**___***Integrity*

Keeps his/her promises

Can be trusted to do the things he/she says.

*Can be relied on to honour his/her commitments (dropped)*

Always keeps his/her words.

**Organizational Culture of Effectiveness** (Sashkin & Rosenbach, 2013).

**___***Managing change*

People are flexible and adaptable when changes are necessary.

People have a clear idea of why and how to proceed throughout the process of change.

People believe they can influence or affect their work place through their ideas and involvement.

**___***Goal achievement*

Individuals and teams have clearly defined goals that relate to the goals or mission of the organization.

Individuals and teams are measured and rewarded according to how well goals are achieved.

Individuals and teams participate in defining specific goals.

**___***Coordinated teamwork*

People believe in teamwork, the “what’s in it for us” approach rather than “what’s in it for me.”

People believe in working together collaboratively, preferring cooperation over completion.

Managers at all levels work together as a team to achieve results for the organization.

**___***Customer orientation*

We give the highest priority and support to meeting the needs of clients and customers and solving their problems.

Our policies and procedures help us to provide the service our customers and clients want and need.

People are always looking for new ways to better serve clients and customers.

**___***Cultural strength*

Everyone knows and understands our objectives and priorities.

People sometimes compromise company policy and procedures to reach operational goals.* (reverse item)

Business decisions are most often made on the basis of facts, not just perceptions or assumptions

**Employee Readiness to Change** (Adapted from Worklife Design, 2008).

When I am impacted by organizational change I am actively involved in shaping the desired future.

When I am affected by organizational change I am involved in identifying possible obstacles

When I am affected by a change in the organization I have the knowledge, skills and abilities necessary to make the change work.
